# Combining BH3-mimetics to target both BCL-2 and MCL1 has potent activity in pre-clinical models of acute myeloid leukemia

**DOI:** 10.1038/s41375-018-0261-3

**Published:** 2018-09-10

**Authors:** Donia M. Moujalled, Giovanna Pomilio, Corina Ghiurau, Adam Ivey, Jessica Salmon, Sewa Rijal, Sarah Macraild, Lan Zhang, Tse-Chieh Teh, Ing-Soo Tiong, Ping Lan, Maia Chanrion, Audrey Claperon, Francesca Rocchetti, Adrien Zichi, Laurence Kraus-Berthier, Youzhen Wang, Ensar Halilovic, Erick Morris, Frédéric Colland, David Segal, David Huang, Andrew W. Roberts, Ana Leticia Maragno, Guillaume Lessene, Olivier Geneste, Andrew H. Wei

**Affiliations:** 1 Australian Centre for Blood Diseases, Monash University, Melbourne, Australia; 2 Department of Clinical Haematology, The Alfred Hospital, Melbourne, Australia; 3 R&D Unit, Institut de Recherches Servier Oncology, Croissy Sur Seine, France; 4 Oncology Disease Area, Novartis Institutes for BioMedical Research, 4056 Basel, Switzerland; 5 Department of Pathology, The Alfred Hospital, Melbourne, Australia; 6 The Walter and Eliza Hall Institute of Medical Research, Parkville, Australia; 7 Oncology Disease Area, Novartis Institutes for BioMedical Research, 250 Massachusetts Avenue, Cambridge, MA 02139 USA; 8 Department of Medical Biology, The University of Melbourne, Parkville, Australia; 9 Department of Clinical Haematology, Royal Melbourne Hospital, Melbourne, Australia; 10 Department of Pharmacology and Therapeutics, The University of Melbourne, Parkville, Australia

**Keywords:** Acute myeloid leukaemia, Translational research

## Abstract

Improving outcomes in acute myeloid leukemia (AML) remains a major clinical challenge. Overexpression of pro-survival BCL-2 family members rendering transformed cells resistant to cytotoxic drugs is a common theme in cancer. Targeting BCL-2 with the BH3-mimetic venetoclax is active in AML when combined with low-dose chemotherapy or hypomethylating agents. We now report the pre-clinical anti-leukemic efficacy of a novel BCL-2 inhibitor S55746, which demonstrates synergistic pro-apoptotic activity in combination with the MCL1 inhibitor S63845. Activity of the combination was caspase and BAX/BAK dependent, superior to combination with standard cytotoxic AML drugs and active against a broad spectrum of poor risk genotypes, including primary samples from patients with chemoresistant AML. Co-targeting BCL-2 and MCL1 was more effective against leukemic, compared to normal hematopoietic progenitors, suggesting a therapeutic window of activity. Finally, S55746 combined with S63845 prolonged survival in xenograft models of AML and suppressed patient-derived leukemia but not normal hematopoietic cells in bone marrow of engrafted mice. In conclusion, a dual BH3-mimetic approach is feasible, highly synergistic, and active in diverse models of human AML. This approach has strong clinical potential to rapidly suppress leukemia, with reduced toxicity to normal hematopoietic precursors compared to chemotherapy.

## Introduction

Acute myeloid leukemia (AML) is a hematopoietic malignancy arising from the transformation of myeloid progenitor cells. Recurrent mutations in over 70 genes characterize the genomic landscape of AML, with each patient harboring a unique spectrum of ancestral, clonal, and sub-clonal populations that evolve over time and in relation to selective pressures exerted by chemotherapy [1, 2]. Although treatment options have remained static for decades, the FDA has recently approved four AML therapies in 2017, including midostaurin, CPX-351, gemtuzumab ozogomycin, and enasidenib [3]. Another promising strategy involves targeting pro-survival activity in AML with BH3-mimetics designed to target BCL-2 and related family members [4]. The most clinically advanced drug is venetoclax, which selectively targets BCL-2 and is approved for clinical use in chronic lymphocytic leukemia [5]. S55746/BCL201 is an orally active, selective, and potent inhibitor of BCL-2 that impairs hematological tumor growth and is currently in phase 1 clinical development [6, 7]. Other BH3-mimetics selectively targeting other pro-survival proteins include A1331852, which inhibits BCL-X_L_ and the recently described MCL1 inhibitor S63845, which our group has shown to be well tolerated in mice and active against a number of malignancies, including a subset of AML at low nanomolar concentrations [8, 9].

Although single agent clinical activity with venetoclax has been modest in AML, clinical responses are increased in combination with either hypomethylating agents or low-dose cytarabine for treatment-naive elderly patients ineligible for intensive chemotherapy [10–12]. In addition to BCL-2, MCL1 is frequently co-expressed in AML and has been shown to play a critical pro-survival role in AML [13]. Chemotherapy enhances anti-leukemic activity by upregulating TP53 and increasing expression of downstream BH3-only members NOXA and PUMA, which are capable of targeting pro-survival proteins not neutralized by venetoclax, such as MCL1 [14, 15]. Venetoclax resistance is associated with upregulation of pro-survival BCL-X_L_ or MCL1, supporting the rationale to target multiple pro-survival proteins simultaneously [13, 14, 16, 17]. We have recently shown that combined targeting of BCL-2 with venetoclax and direct inhibition of MCL1 with a lentiviral BH3-expressing vector was highly effective in producing prolonged remissions in xenograft models of AML [14]. Although synergy between BH3-mimetics targeting BCL-2 and MCL1 has been previously reported in AML, prior MCL1 inhibitors have not been suitable for clinical development [18].

The potential to clinically target MCL1 was realized by development of the potent and selective small molecule inhibitor S63845 [8]. Although we previously showed that pharmacological targeting of MCL1 was tolerated by normal human CD34 + precursors, the tolerability of targeting multiple pro-survival proteins using BH3-mimetics has not been reported [18]. We now show that simultaneous inhibition of both MCL1 (with S63845) and BCL-2 (with venetoclax or S55746) is highly synergistic in AML and extends low nanomolar activity with the combination to approximately half the primary AML samples tested, spanning a broad spectrum of cytogenetic and molecular profiles. S55746 in combination with S63845 was also active against chemotherapy relapsed and refractory AML samples, indicating therapeutic potential for patients with chemoresistant disease. Synergy with S63845 was stronger in combination with BCL-2 inhibitors than with cytotoxic or hypomethylating agents. Simultaneous BH3-mimetic targeting of BCL-2 and MCL1 produced rapid and durable remissions in cell line xenograft models and bone marrow cytoreductions in patient-derived xenograft (PDX) models of AML. Despite robust activity against leukemic progenitors, pharmacologic inhibition of BCL-2/MCL1 was less toxic to normal CD34 + progenitors than standard cytotoxic drugs. Our results support the clinical development of a novel, non-chemotherapy based approach, combining BH3-mimetics to target both BCL-2 and MCL1 in patients with AML.

## Materials and methods

### AML cell lines

MV4;11, MOLM-13, PL-21, ML-2, Nomo-1, THP-1, EOL-1, Kasumi-1 (from ATCC) were cultured in RPMI (GIBCO) supplemented with FBS 10% (v/v); HL-60, KG1, KG1a were cultured in IMDM (GIBCO) supplemented with FBS 20% (v/v); and OCI-AML3 in MEM alpha (GIBCO) supplemented with FBS 20% (v/v). In addition, all media contained penicillin (100 IU/ml), streptomycin (100 µg/ml), and L-glutamine (2 mM). All cell lines were determined to be free of mycoplasma contamination.

### Drugs, cell viability, and synergy assays

S55746 and S63845 were provided by Servier Laboratories. A1331852 was synthesized as previously described [9]. Venetoclax was purchased from Active Biochem (Cat. No. A-1231). Decitabine was purchased from Abcam (Cat. No. ab120842), Idarubicin was purchased from Selleckchem (Cat. No. S1228), Cytarabine was purchased from Hospira. Bone marrow samples from patients with AML and donor CD34 + cells were collected after informed consent in accordance with guidelines approved by The Alfred and Royal Melbourne Hospital human research ethics committees. Ficoll-purified and red cell depleted AML cells were plated in RPMI and 15% FBS at 2.5 × 10^5^ cells/mL and drugs tested over a 5-log concentration range. After 48 h, cell viability was determined by FACS analysis of cellular exclusion of SYTOX Blue Dead Cell Stain (Life Technologies Cat No S34857) using an LSR-Fortessa (BD) [19, 20]. FACS data was analyzed using the FlowJo software. GraphPad Prism software was used to calculate drug concentrations causing 50% lethality (LC_50_).

For the larger scale drug synergy assays, cells were seeded and treated with nine 2-fold serial dilutions of each compound dispensed into cell assay plates. Single agent IC_50_’s were calculated using standard four-parametric curve fitting. IC_50_ was defined as the compound concentration at which the Cell Titer Glo (CTG) signal was reduced to 50% of that measured for the vehicle (DMSO) control. In order to analyze the activity of the compounds in combination, the cells were seeded and treated with seven or eight 3.16-fold serial dilutions of each compound dispensed, either individually or in all possible permutations in a checkerboard fashion. Effects of the single agents, as well as their checkerboard combinations on cell viability were assessed after three days of incubation at 37 °C/5% CO_2_ using CTG at 75 μL reagent/well. Two independent experiments, each one performed in duplicate, were performed. Luminescence was quantified on a multipurpose plate reader. Potential synergistic interactions between compound combinations were assessed using the Loewe additivity model (Chalice^TM^ Bioinformatics Software available in Horizon website) and reported as the Synergy Score [20]. Cell viability assays for LC_50_ determination and colony forming unit (CFU) assays have been previously described [18].

### Targeted sequencing

Genomic DNA libraries were prepared using either a custom Haloplex (Agilent) panel, as previously described [21], or the TruSight Myeloid Sequencing Panel (Illumina) according to the manufacturer’s instructions. Sequencing was carried out on the Illumina NextSeq 500 platform with 150-bp paired-end reads. The resulting FASTQ files were aligned using the TruSeq Amplicon App (version 3.0.0) on BaseSpace (Illumina) and variants called using the Somatic Variant Caller (Illumina). The vcf files generated were filtered in Variant Studio (Illumina) and further verified using the Integrative Genomics Viewer (Broad Institute).

### Mouse AML xenograft models

All studies in mice were performed under the institutional guidelines approved by the Alfred Medical Research and Education Precinct Animal Ethics Committee. MV4;11 cells transduced with the luciferase reporter (pLUC2 promega) were intravenously injected at 1 × 10^5^ cells into 6-week old female and male irradiated (100 Rad) non-obese diabetic/severe combined immunodeficient (NOD/SCID/IL2rγ^null^) mice as previously described [21]. Engraftment was measured on day 7 (MV4;11) or day 38 (OCI-AML3) by quantifying the percentage of hCD45 + cells in the peripheral blood (PB) by flow cytometry and by IVIS imaging of bioluminescent MV4;11 or OCI-AML3 cells. After detectable engraftment, mice were gavaged with S55746 (200 µL 100 mg/kg) dissolved in PEG400 (Sigma), absolute ethanol (Sigma), and distilled H_2_0 at a ratio of 40:10:50 or S63845 (200 µL 25 mg/kg) twice weekly or weekly IV dissolved in 50% 2-hydroxypropyl)-β-cyclodextrin (Sigma) and 50% 50 mM HCL or the drug combination, or vehicle. Blood counts were determined using a hematology analyzer (BioRad, Gladesville, NSW). Bioluminescent imaging was performed using the Caliper IVIS Lumina III XR imaging system. Mice were anesthetized with isoflurane, injected intraperitoneally with 100 µL of 125 mg/kg luciferin (Perkin Elmer, Springvale, VIC), and BLI performed 5 minutes later.

### Patient-derived xenograft models of AML

To establish mouse models of primary patient AML, 1 × 10^6^ leukemic blasts were injected into 6-week old female NOD-IL2Rcγ^null^ (NRG-SG3) mice (The Jackson Laboratory, Bar Harbor, ME, USA) via tail-vein injection and animals monitored for leukemia progression using flow cytometric analysis of peripheral blood for hCD45 + cells. hCD45 + cell counts in the bone marrow from the femurs of euthanized animals were used to determine the extent of leukemia infiltration. Bone marrow cells were extracted by flushing femurs in PBS supplemented with 2% fetal bovine serum. To determine the efficacy of S55746 and S63845, mice were gavaged daily with S55746 (100 mg/kg) for 5 days or received S63845 25 mg/kg twice weekly IV. Drug efficacy was determined by flow cytometric analysis of hCD45 + cells in bone marrow isolated from flushed femurs. Sternums and spleens were fixed in formalin, sectioned, and stained with hematoxylin and eosin or anti-hCD45 to assess leukemic burden and cellularity.

For additional methods refer to Supplementary Information.

## Results

### Combined targeting of BCL-2 and MCL1 induces potent and synergistic apoptosis in primary AML samples

**Fig. 1:**
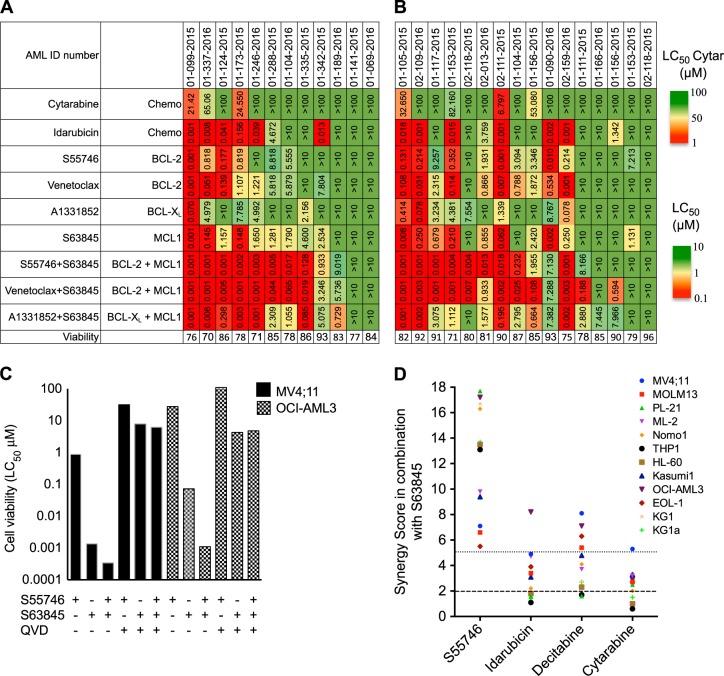
**Interrogating pro-survival dependency in AML using BH3-mimetic drugs alone and in combination**. The sensitivity (LC_50_) of freshly derived primary samples to BH3-mimetics alone, or in equimolar combination, relative to chemotherapy (cytarabine and idarubicin) after 48 h exposure. Samples are separated according to whether patients were **a** chemotherapy naive or had **b** relapsed and refractory AML. The control cell viability of each AML sample after 48 h in DMSO is shown. The upper concentration of cytarabine tested was 100 µM and for other drugs 10 µM. A color bar grading the LC_50_ values for each drug in the heat map is shown. **c** MV4;11 and OCI-AML3 cells were treated with indicated drugs and the LC_50_ at 16 h determined. Where indicated, cells were pre-incubated with the caspase inhibitor QVD (50 μM) for 1 h prior to addition of the other drugs. **d** Synergistic interactions between S63845 combined with standard anti-leukemic drugs were assessed using an Excess Inhibition matrix according to the Loewe additivity model. Synergy scores (SS) are represented as the mean of *n* = 2; each experiment being performed in duplicate (SS = 0 represents an additive effect, SS > 2 represents synergy; hashed line and SS > 5 represents strong synergy; dotted line)

We first compared the pro-survival dependency of primary AML samples treated with a panel of potent and selective inhibitors of MCL1 (S63845), BCL-2 (ABT-199/venetoclax or S55746/BCL201), or BCL-X_L_ (A1331852). Comparison was made with idarubicin and cytarabine, drugs commonly used in the treatment of AML. Freshly derived primary AML samples were studied if the viability of the cells in control media plus DMSO after 48 h was at least 70%. Primary samples were derived from patients with treatment naive (Fig. 1a) or chemotherapy relapsed/refractory (Fig. 1b) AML.

Consistent with previously published work, a minor proportion of primary AML cases appeared highly sensitive (LC_50_ < 100 nM) to BCL-2 (venetoclax or S55746), or MCL1 (S63845) targeting alone [8]. Fewer primary samples were sensitive to the BCL-X_L_ inhibitor A1331852 (Fig. 1a, b). The activities of the BCL-2 inhibitors venetoclax and S55746 appeared highly correlated (Fig. 1a, b and Supp. Figure 1A). In contrast to modest single agent activity observed from targeting BCL-2, BCL-X_L_, or MCL1 alone, marked anti-leukemic activity (LC_50_ sensitivity < 100 nM) was apparent in > 50% of the primary AML samples when treated simultaneously with equimolar concentrations of BH3-mimetics targeting BCL-2 and MCL1 (Fig. 1a, b). Similar activity was observed for S63845 combined with either venetoclax or the BCL-2 inhibitor S55746. In contrast, combined inhibition of pro-survival BCL-X_L_ and MCL1 was less effective, suggesting that in the majority of AML cases, pro-survival function in AML was predominantly mediated by BCL-2 and MCL1. Co-targeting BCL-2 and MCL1 was also efficacious (LC_50_ < 100 nM) in a subset of primary AML samples resistant to the anthracycline drug idarubicin (LC_50_ > 1 μM; Fig. 1a, b). The sensitivity of AML samples to combined S55746 and S63845 was similar among samples from patients treatment naïve or relapsed/refractory to prior chemotherapy (Fig. 1a, b and Supp. Figure 1B). Leukemic cell death from combined S55746 and S63845 therapy was caspase (Fig. 1c and Supp. Figure 1C, D) and BAX/BAK dependent (Supp. Figure 1E, F). In summary, our findings suggest that BCL-2 and MCL1 are key determinants of pro-survival activity in both treatment naive and chemoresistant AML and that simultaneous targeting of both proteins appears necessary for optimal pro-apoptotic activity [14].

### Anti-leukemic synergy of S63845 combined with BCL-2 inhibition compared to standard drugs

Comparative drug synergy experiments between the MCL1 inhibitor S63845 and standard drugs was not possible using primary AML samples, due to insufficient cell numbers. Therefore, a series of 12 AML cell lines was employed to assess the activity of S63845 in combination with S55746, cytotoxic drugs (idarubicin, cytarabine), or the hypomethylating agent decitabine (Fig. 1d). Synergistic interactions were assessed using the Loewe additivity model to derive Synergy Scores (SS); with a SS > 2 indicating synergy and a SS > 5 strong synergy [22]. Single agent LC_50_ and representative effect and synergy matrices for the cell line OCI-AML3 treated with S63845 and S55746 are shown (Supp. Figure 2A,B). The strongest synergistic interaction was observed between S63845 and S55746 (SS > 5 in 12/12 AML cell lines tested). In comparison, for S63845 in combination with decitabine, synergy scores > 5 were observed in 4 out of 12 AML cell lines tested, whereas for S63845 combined with cytotoxic drugs idarubicin or cytarabine, most synergy scores were < 5 (Fig. 1d).

### Combined targeting of BCL-2 and MCL1 is efficacious in poor risk AML

**Fig. 2:**
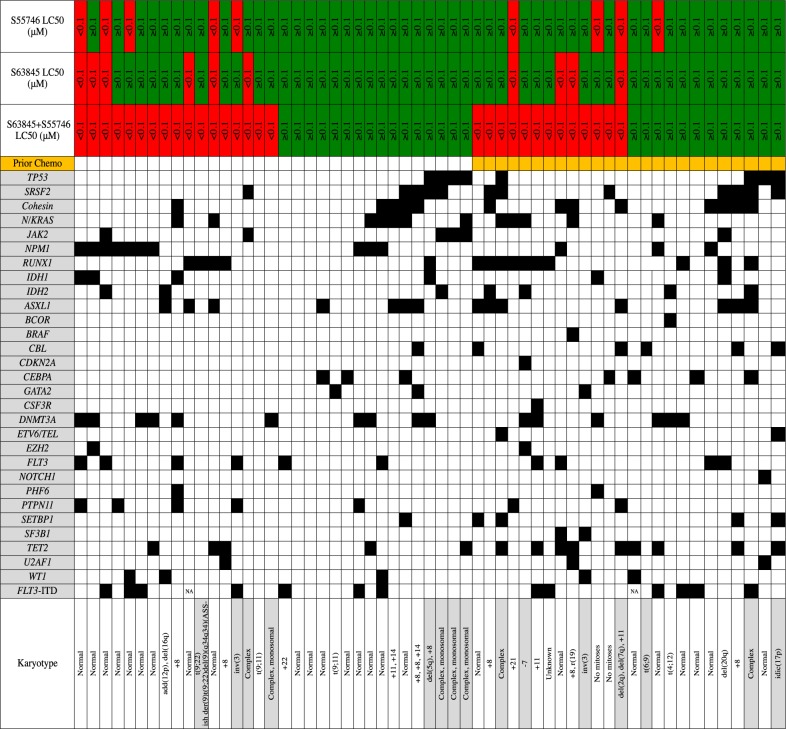
**Mutation profiling of primary AML samples and sensitivity to BCL-2/MCL1 targeting.** Sensitivity to combined S55746/S63845 is shown, with sensitive samples (LC_50_ < 100 nM) colored red and resistant cases (LC_50_ > 100 nM) green. Prior patient resistance to chemotherapy is indicated by orange colored boxes. Mutation presence is indicated by filled black boxes for each sample. Cytogenetic abnormalities are summarized, with gray shading indicating adverse-risk karyotype. Missing FLT3-ITD values are indicated (NA)

Poor survival outcomes in AML are associated with adverse-risk chromosomal lesions (complex karyotype, aneuploidy, t(6;9), inv(3), MLL-fusions), mutations involving *TP53* or the RNA splicing/chromatin machinery (*RUNX1, ASXL1, BCOR, STAG2, EZH2, SRSF2, SF3B1, U2AF1, ZRSR2* or *MLL*^PTD^) [1, 23]. Therefore, the cytoreductive effect of dual BCL-2 and MCL1 targeting was explored across treatment naïve and chemotherapy relapsed/refractory AML samples characterized for cytogenetic abnormalities and recurrent mutations using a 54-gene targeted exome panel (Fig. 2) [24]. Sensitivity to combined BCL-2/MCL1 targeting (LC_50_ < 100 nM) was observed across a broad spectrum of AML cases, including those with recognized poor risk genomic features, such as mutated *RUNX1* (11/15 cases sensitive), *DNMT3A* (10/16 cases sensitive) or *ASXL1* (7/14 cases sensitive)(Fig. 2) [1]. A notable observation was potential resistance to combined BCL-2/MCL1 targeting (LC_50_ > 100 nM) among cases harboring mutant *TP53* (7/8 resistant). As overall numbers were limited, an expanded sample set will be required to confirm this preliminary association.

### Leukemic stem and progenitor cells were more sensitive to BCL-2/MCL1 targeting than normal CD34+progenitors

**Fig. 3:**
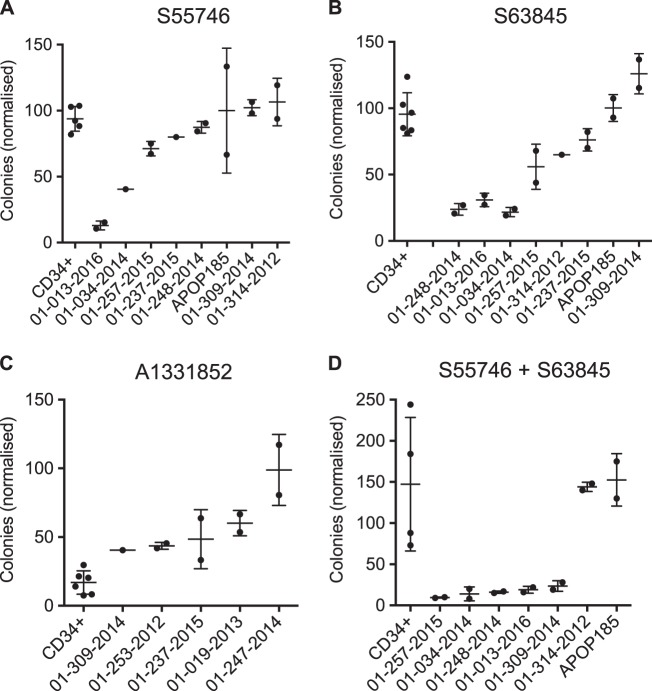
**Comparison of the effects of BCL-2 and MCL-1 targeting on leukemic compared to normal progenitor function**. Suppression of clonogenic activity by **a** S55746 **b** S63845 **c** A1331852, or **d** combined S55746/S63845. Between 10^4^–10^5^ primary AML or normal donor CD34 + cells (3–4 donors) were plated in 0.6% agar supplemented with GM-CSF, IL-3, SCF, and EPO and cultured for 2–3 weeks at 37 °C with 100 nM of each BH3-mimetic. Colonies were enumerated by GelCount^TM^ and normalized to the number of colonies from primary AML or CD34 + cells treated with DMSO. AML colony numbers (as a % normalized to DMSO) are shown with error bars indicating mean + /− 1 s.d. of each AML sample performed in duplicate. In the CD34 + column for each drug, colony numbers (as a % normalized to DMSO) from duplicate experiments from 2 or 3 donors are pooled and shown. To facilitate comparison, Fig. 3b includes data from our prior publication [8] with the addition of two new samples

We have previously shown that a small subset of primary AML samples are sensitive to the MCL1 inhibitor S63845 in long-term clonogenic assays, with less toxicity observed in normal donor CD34 + cells, highlighting a therapeutic window for MCL1 targeting leukemic progenitors [8]. In contrast, cytotoxic drugs cytarabine, and idarubicin were toxic to both leukemic and normal progenitors, consistent with the known severe myelosuppressive effects associated with these drugs [8]. We now show that combined targeting of BCL-2 and MCL1 dramatically enhances activity against AML progenitors, compared to either drug given alone (Fig. 3a, b, d). The combination suppressed clonogenic activity (by > 50%) in 5/7 primary AML cases tested, with minimal impact on the clonogenic growth of normal CD34 + cells (Fig. 3d). In contrast, BCL-2 or MCL1 targeting alone only suppressed clonogenic activity in 2–3/8 cases each. Interestingly, the selective BCL-X_L_ inhibitor A1331852 was toxic to both leukemic and normal progenitors in clonogenic assays (Fig. 3c). These findings demonstrate that combined BCL-2/MCL1 targeting has the best therapeutic index for suppressing human leukemic stem and progenitor cells.

### Combined therapy with S55746 and S63845 has potent anti-leukemic activity in vivo

**Fig. 4:**
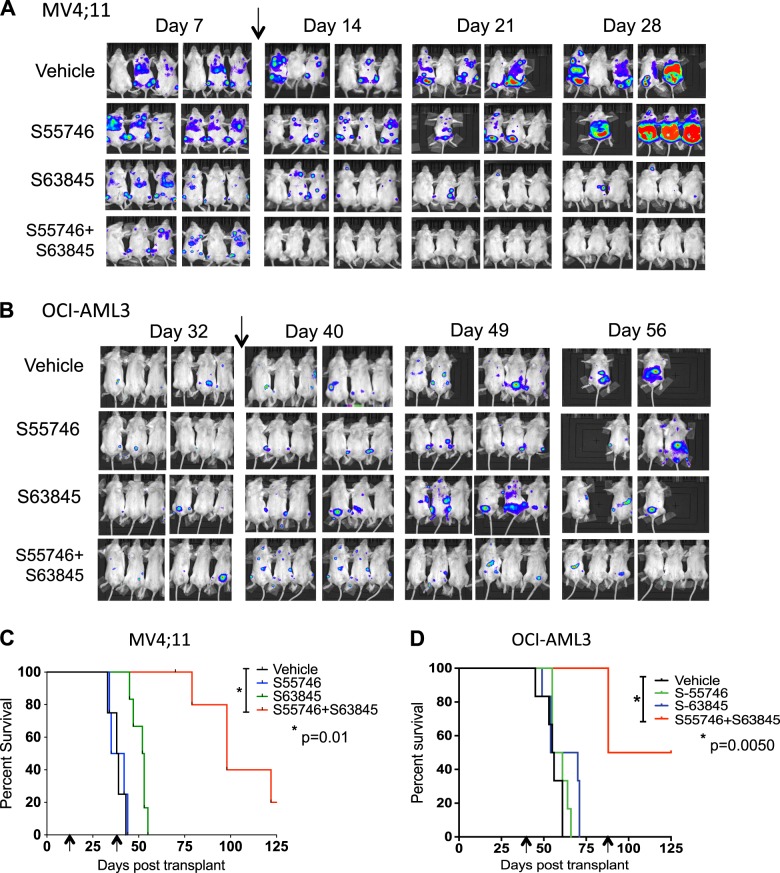
**Combined targeting of BCL-2 and MCL1 improves the survival of mice xenografted with human AML** (**a**) 24 irradiated NSG mice were transplanted with 10^5^ human MV4;11 cells transduced with a luciferase reporter construct. AML engraftment was confirmed by bioluminescence imaging on day 7. On day 10 (arrow), mice were divided into treatment groups of six mice and treated with (i) vehicle, (ii) S55746 100 mg/kg by oral gavage daily (5 days/week for 4 weeks), (iii) S63845 25 mg/kg IV twice weekly for 4 weeks, or (iv) combination S55746/S63845 for 4 weeks, with treatment ending on day 35. **b** Similar experiment as in (A) using OCI-AML3 cells transduced with a luciferase reporter. Engraftment was confirmed on day 32. Treatment commenced on day 38 (arrow) post-transplant for a total of 7 weeks. **c** NSG mice engrafted with human MV4;11 and treated as in (**a**) and followed for Kaplan–Meier (KM) survival (ethical endpoints) showing that combined treatment with S55746/S63845 resulted in significantly longer survival than vehicle control (arrows show start and end of treatment). **d** KM survival of NSG mice engrafted with human OCI-AML3 cells and treated as in (**a**) showing that combined treatment with S55746/S63845 resulted in significantly longer survival than vehicle control (arrows show start and end of treatment)

Our previous work showed that mice engrafted with MV4;11 and OCI-AML3 cells could achieve sustained remissions if BCL-2 and MCL1 were concomitantly targeted [14]. This previous study employed venetoclax to target BCL-2 in combination with a lentiviral BIM-BH3 vector designed to conditionally target MCL1 in vivo [14]. The limitation of this experiment was that MCL1 was continuously targeted by the lentivirus, which does not reflect how a drug delivered intermittently would work. We therefore sought to determine whether pharmacological targeting of BCL-2 and MCL1 using a small molecule approach could similarly enhance survival in animal model systems of AML. Preliminary in vitro studies confirmed the efficacy of combined BCL-2 and MCL1 targeting with S55746 and S63845, respectively, to enhance caspase–dependent pro-apoptotic activity of both MV4;11 and OCI-AML3 cell lines (Fig. 1c). To demonstrate synergistic activity in vivo, immunodeficient NSG mice were engrafted with luciferase-labeled MV4;11 or OCI-AML3 cells and monitored by bioluminescence imaging (BLI) for tumor development in vivo. Whole body imaging identified foci of engrafted bioluminescent-avid MV4;11 tumors by day 7 and OCI-AML3 tumors by day 32 post-transplant, respectively (Fig. 4a, b). Mouse cohorts with established leukemia were then treated with either S55746, S63845, or the combination of both drugs. In mice xenografted with MV4;11 AML, eradication of bioluminescent-avid disease was most effective with combined S55746 and S63845 (Fig. 4a). Although partial early suppression of MV4;11 leukemia was achieved by S55746 or S63845 monotherapy, this did not translate into significant improvements in long-term survival (Fig. 4c). Combined S55746/S63845, however, increased median survival of established AML over three-fold in the MV4;11 model, even though treatment ceased on day 35 (Fig. 4c).

In the OCI-AML3 model, combined S55746/S63845 was also more effective at suppressing AML than either drug alone as assessed by BLI (Fig. 4b). In contrast to the MV4;11 model, residual BLI-avidity for OCI-AML3 tumor was still evident after 4 weeks of treatment with S55746/S63845 (Fig. 4b). OCI-AML3 cells are more resistant in vitro and express higher levels of MCL1 than MV4;11 cells (Figure Suppl. Figure 2A and not shown). Therefore, treatment commenced on day 38 and was continued for 7 weeks. This resulted in 50% survival by day 125 in mice treated with combined S55746 and S63845, compared to no surviving mice beyond day 75 in vehicle and single agent arms (Fig. 4d).

### Combination therapy with S55746/S63845 suppresses patient-derived AML in vivo

**Fig. 5:**
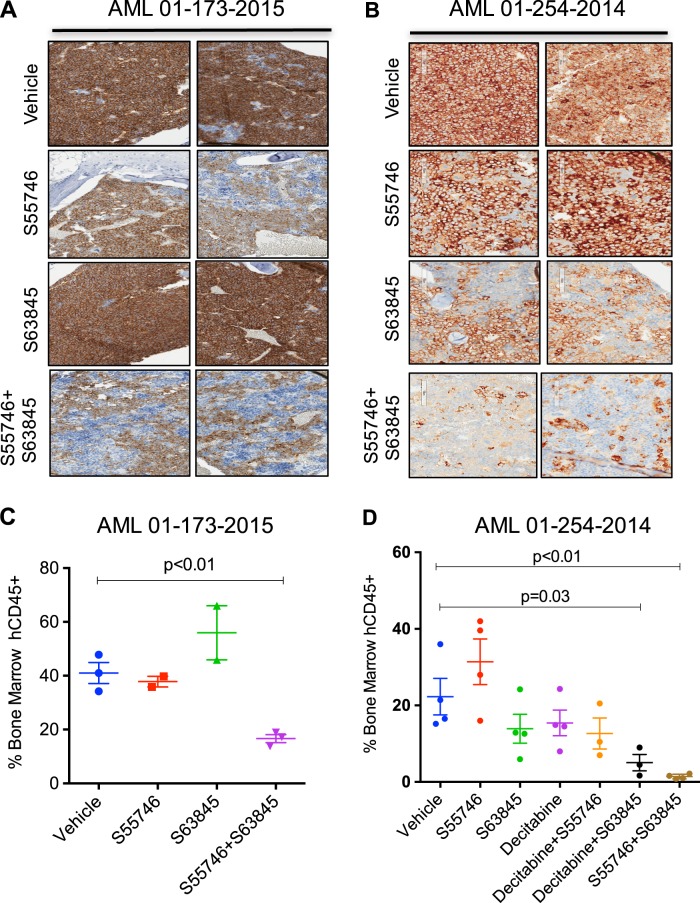
**Combined S55746 and S63845 suppresses leukemia in vivo in NRG-SG3 patient-derived xenograft models of AML.** **a** Irradiated NRG-SG3 mice were transplanted with 10^6^ primary AML cells (AML01-173-2015). Engraftment was confirmed at 6 weeks by detection of hCD45 in peripheral blood. Cohorts of 2–4 mice per group were then treated with (i) vehicle (d1–5), (ii) S55746 100 mg/kg days 1–5 by gavage, (iii) S63845 25 mg/kg IV on days 2 and 4), or (iv) S55746 combined with S63845. Mice were euthanized on day 8 and immunohistological analysis for hCD45 + performed on sternal bone marrow for infiltration by AML cells captured at ×100 magnification using the Aperio ScanScope. Two representative cases from each cohort are shown. **b** Similar experiment as in (**a**) with NRG-SG3 mice engrafted with AML 01-254-2014 and treated with (i) vehicle, (ii) S55746, (iii) S63845, or (iv) S55746 combined with S63845. Flow cytometric analysis of flushed femurs on day 8 showing the percentage of human CD45 + blasts from AML 01-173-2015 (**c**) after (i) vehicle, (ii) S55746, (iii) S63845, and (iv) S55746 combined with S63845. Similar experiment in **d** using AML 01-254-2014 showing the effects of (i) vehicle, (ii) S55746 (iii) S63845 (iv) S55746 combined with S63845, (v) decitabine 0.4 mg/kg IV days 1–5, (vi) decitabine plus S55746, or (vii) decitabine plus S63845. In (**c**, **d**) mean, + /- 1 s.d. from individual mouse values are shown

To examine the potential for dual BCL-2 and MCL1 targeted therapy to suppress patient-derived AML in vivo, primary AML samples were xenografted into immunodeficient NOD.*Rag1*^*−/−*^*;γc*^null^ (NRG)-SG3 mice, which have been transgenically modified to express human SCF, GM-CSF, and IL3 in vivo, to enhance engraftment of human AML blasts [25, 26]. Strikingly, in two independent PDX models of AML (AML 01-254-2014 and AML01-173-2015), S55746/S63845 led to cytoreduction of AML after only 5 days of treatment as assessed by immunohistological staining of mouse sternums (Fig. 5a, b) or flow cytometric enumeration of human CD45 + blasts from flushed femurs (Fig. 5c, d). In AML 01-254-2014, the anti-leukemic effect of dual BCL-2/MCL1 targeting appeared more effective than a 5-day schedule of decitabine and comparable to the effect of decitabine in combination with BH3-mimetics targeting either BCL-2 or MCL1 (Fig. 5d).

### Combined BCL-2 and MCL1 targeting was less toxic to engrafted normal human hematopoiesis than chemotherapy in vivo

**Fig. 6:**
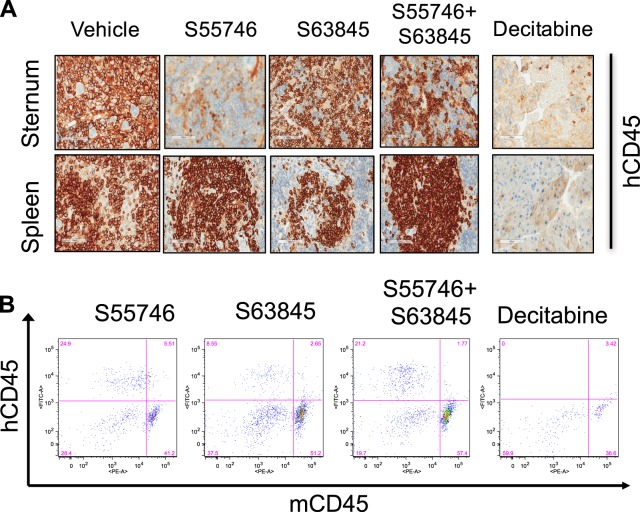
Combined S63845 and S55746 did not affect normal hematopoietic cell function. **a** Irradiated NSG mice were transplanted with 10^5^ normal donor CD34 + progenitor cells. Following detectable engraftment in peripheral blood at week 20, mice were treated with (i) S55746 100 mg/kg/d by oral gavage (days 1–5), (ii) S63845 25 mg/kg IV (days 1 and 4), (iii) combination S55746/S63845, or (iv) decitabine 0.4 mg/kg intraperitoneally (days 1–5). Mice were euthanized on day 8 and immunohistological hCD45 + analysis performed on sternal bone marrow and spleen sections captured at ×100 magnification using the Aperio ScanScope. Due to limited availability of donor CD34+cells, 2 mice were engrafted and treated in arms i–iii and one mouse in arm iv; representative images are shown with additional examples in Suppl Fig. 3. **b** Representative flow cytometric dot plots of flushed femurs from mice treated as indicated in (**a**) are shown displaying the proportion of marrow cells expressing human versus mouse CD45

Having demonstrated the potential for dual BH3-mimetic targeting of BCL-2 and MCL1 to rapidly induce remissions and extend the survival of mice xenografted with AML, we next examined the tolerance of normal hematopoietic cells to combined S55746/S63845 therapy. Prior published conditional *Mcl1* gene knockout studies suggested that long-term *Mcl1* ablation was poorly tolerated by mouse hematopoietic cells [27]. The MCL1 inhibitor S63845 targets human MCL1 with 6-fold higher affinity than mouse Mcl1 [8]. We therefore sought to verify the effect of pharmacologic targeting of both MCL1 and BCL-2 on human hematopoietic function by engrafting NSG mice with CD34 + cells from a normal human donor. After confirmation of engraftment, mice were treated with S55746 and S63845 over a five-day period. In contrast to marked suppression of AML in vivo (Fig. 5), immunohistological staining of engrafted donor CD34 + cells in sternal bone marrow (Fig. 6a and Suppl Fig. 3) and flow cytometric enumeration of flushed femurs (Fig. 6b) showed minimal impact of combined S55746/S63845 treatment on normal CD45 + cells. In contrast, decitabine therapy was toxic (Fig. 6a, b). Collectively, these results suggest that targeting both BCL2 and MCL1 using small molecule inhibitors to suppress human AML in vivo may have less severe adverse effects on normal bone marrow hematopoietic function than standard cytotoxic drugs.

## Discussion

Since its inception in the 1970’s, combined cytarabine/anthracycline or “7 + 3” chemotherapy has been an enduring constant in the therapy of AML. Our current work highlights several new findings of translational importance for patients with AML. We demonstrate for the first time that small molecule BH3-mimetics directly targeting BCL-2 and MCL1 can potently suppress human AML, with limited toxicity to normal human hematopoietic progenitors, potentially overcoming one of the major limitations from the use of cytotoxic drugs. Furthermore, primary AML blasts were more sensitive to combined MCL1/BCL-2 than to combined MCL1/BCL-X_L_ targeting. This avoids undesirable on-target thrombocytopenia that may result from BCL-X_L_ inhibition [28]. Our studies also demonstrate the anti-leukemic activity of a new BCL-2 inhibitor (S55746), in combination with the recently described MCL1 inhibitor S63845. The translational potential of these findings in AML are high, as both S55746 (NCT02920541) and the clinical MCL1 inhibitor derivative S64315 (NCT02979366) are both currently undergoing phase 1 clinical evaluation in human AML.

Although venetoclax has previously been shown to synergistically enhance the activity of the MCL1 inhibitor A-1210477, the latter was only been shown to be effective in vitro and against AML cell lines at micromolar concentrations [29]. In contrast, the current work shows that combined S55746 and S63845 has low nanomolar activity across a broad spectrum of AML genotypes, including primary samples from patients with adverse genetic risk and chemoresistant AML. A proportion of primary AML samples remained resistant to BCL-2/MCL1 targeting (Fig. 2). Our gene mutation profiling studies showed enrichment of samples with *TP53* mutation in this sub-group. The mechanistic basis of this resistance remains to be determined. Possible causes include expression of non-targeted pro-survival factors, such as BFL-1 or BCL-X_L_, the requirement to target multiple pro-survival proteins simultaneously (e.g., BCL-2/BCL-X_L_/MCL1) or defective expression of downstream BAX or BAK. Examination of these possibilities will be the subject of future research.

Prior to this study, the feasibility of targeting both BCL-2 and MCL1, even with pharmacological inhibitors, remained uncertain. Previous lineage-specific deletion models indicated potential risk to cardiac [30, 31], granulocyte/hematopoietic [27, 32–34], lymphocyte/thymocyte [35, 36], neuronal [37], and liver function [38, 39] as a consequence of long-term MCL1 ablation. We have recently shown that an intermittent weekly or twice weekly schedule of a potent short-acting pharmacological inhibitor of MCL1 is well tolerated in animal models and active against a range of cancers in vivo, including AML [8]. The short protein half-life of MCL1 permits rapid regeneration in critical organs, potentially supporting physiological tolerance to short-term S63845 exposure [40]. Until now, pulsatile inhibition of BCL-2 and MCL1 mimicking a drug-like effect has not been possible using genetically engineered approaches. The availability of potent and selective small molecule inhibitors of BCL-2, BCL-X_L_, and MCL1 enables determination of which pro-survival proteins are important for the survival of normal, as well as malignant tissues, both in vitro and in vivo. Importantly, the sensitivity of intact cells to BH3-mimetics can be measured quantitatively, utilizing compounds intended for clinical use.

In conclusion, our studies using S55746 and S63845 provide proof-of-concept demonstration that targeting BCL-2 and MCL1 simultaneously can lead to rapid suppression of diverse AML subtypes, with limited toxicity to normal human bone marrow cells, thereby providing strong rationale for further clinical development.

## Electronic supplementary material

Moujalled et al_Supplementary information
