# Supplementary material for: Combining BH3-mimetics to target both BCL-2 and MCL1 has potent activity in pre-clinical models of acute myeloid leukemia
Source: Leukemia. 2018 Sep 10;33(4):905–17. doi: 10.1038/s41375-018-0261-3 (PMC6484700; doi:10.1038/s41375-018-0261-3)
Supplement: Supplementary file 1 — Moujalled et al_Supplementary information [file 41375_2018_261_MOESM1_ESM.docx]

**Supplementary Information**

**Materials and Methods**

***Immunohistological Studies***

Mouse sternums and spleens were harvested and fixed in 3.7% formalin for a minimum of 24h before decalcification in phosphate-buffered formalin (3.7% formalin, pH 7.4) containing 20% EDTA for 48h, then embedded in paraffin and sectioned. For immunohistological staining of human CD45, slides were prepared using standard procedures and stained with anti-human CD45 mAb (Dako, Cat. No. M0701) in a Dako Autostainer Plus. Slides were imaged and annotated using an Aperio ScanScope and software system (Leica Biosystems).

***CRISPR/Cas9 genome editing***

THP-1 *BAK*^-/-^*BAX*^-/-^ cells were generated by serial transduction with lentiviral particles (Sigma Aldrich), at a multiplicity of infection (MOI) of 10, in the presence of 8µg/ml of polybrene. First, cells were transduced with a Cas-9 expressing lentivirus (EF1a-Cas9-2A Neomycin), selected with neomycin (800µg/ml, Gibco) and sorted as single cells for the isolation of clones. One clone was transduced with a lentivirus (pLV-U6g-EGFP) expressing sgRNA to *BAK1* (5' GCATGAAGTCGACCACGAAG 3'), re-cloned on the base of GFP reporter gene expression and verified by western blot. To generate the *BAK*^-/-^*BAX*^-/-^ THP-1 clone, a BAK-deficient clone was transduced with a lentivirus (pLV-U6g-EPuro) expressing sgRNA to *BAX* (5' CTGCAGGATGATTGCCGCCG 3'), selected with puromycin (1µg/ml), cloned and verified by western blot.

***Statistics***

For mouse experiments, mice were randomized to treatment cohorts by the coin-toss method.

**Supplementary Figures**

**Figure S1.**

1. **Comparison of the activity of BCL-2 inhibitors, venetoclax or S55746, against primary AML samples.** AML cells were freshly harvested from patient bone marrow and isolated by ficoll gradient-separation and red cell lysis followed by incubation with venetoclax or S55746 for 48 h. The LC_50_ for venetoclax and S55746 was plotted for each sample (n = 49) and the degree of correlation (r) shown.
2. **Comparison of combined S55746/S63845 in primary samples from patients with treatment naïve or relapsed/refractory AML.** LC_50_ (*μ*M) values after treatment with S55746, S63845 or combined S55746/S63845 for 48 h on primary samples from patients with AML that were either treatment naïve or relapsed/refractory to prior chemotherapy. The percentage of samples with an LC_50_ < 0.1 *μ*M (dotted line) is shown.
3. **Enhanced AML cell death induced by targeting BCL-2 and MCL1 is caspase- dependent.** Primary AML samples were treated with indicated drugs for 48 h. Caspase dependency of BH3-mimetic activity in primary AML patient samples was demonstrated by showing reversibility of cell death by pre-incubation with the caspase inhibitor QVD (50 *μ*M) for 1h, followed by addition of S55746, S63845, or the combination (1 nM to 10 *μ*M) for 16 h and LC_50_ determined.
4. As in (C) with primary AML sample AML 01-311-2014
5. **BAK and BAX/BAX deletion** following CRISPR/CAS9 genomic editing in a THP-1 cell line was verified by western blot
6. **Synergy matrix for THP-1 and THP-1 *BAK^-/-^/BAX^-/-^* cells treated with S63845 and S55746.** Cells were treated and analysed as described in methods. The double knockout THP-1 *BAK^-/-^/BAX^-/-^* cells were resistant to combined S63845 and S55746 treatment.

**Figure S2.**

1. **IC_50_ sensitivity of AML cell lines to experimental drugs.** Indicated cell lines were treated with serial dilutions of each compound and IC_50_ (nM of compound at which the CellTiterGlo (CTG) signal is reduced to 50% of that measured for the vehicle control) calculated using standard four-parametric curve fitting.
2. **Effect and synergy matrix for the OCI-AML3 cell line treated with S63845 and S55746**. Cells were treated with serial dilutions of each compound either individually or in various permutations in a checkerboard fashion. Cell viability was assessed 72 h later using CTG. Effect matrix (left panel) showing the % cell growth inhibition compared to vehicle control (0 = no inhibition of cell growth and 100 = total inhibition of cell growth). In the synergy matrix (right panel) the values represent the extent of cell growth inhibition in excess of the theoretical additivity based on single agent activities of S63845 and S55746 using the Loewe additivity model.

**Figure S3. Combined S63845 and S55746 did not affect normal hematopoietic cell function- additional examples for figure 6**

**Table S1. Clinical information for AML samples**
